# Identification of a de novo case of *COL5A1*‐related Ehlers‐Danlos syndrome in an infant in the West Indies leading to improved targeted clinical care

**DOI:** 10.1002/ccr3.1873

**Published:** 2018-10-15

**Authors:** Amr Wardeh, Tyson Jackson, Beverly Nelson, Carl Ernst, Jean‐François Théroux, Walla Al‐Hertani, Andrew K. Sobering, Mary C. Maj

**Affiliations:** ^1^ Department of Biochemistry St. George’s University St. George’s Grenada; ^2^ Pediatrics Ward Grenada General Hospital St. George's Grenada; ^3^ Department of Psychiatry McGill University Montreal Québec Canada; ^4^ Department of Medical Genetics, Cummings School of Medicine University of Calgary, Alberta Children's Hospital Calgary Alberta Canada

**Keywords:** *COL5A1*, developmental delay, Ehlers‐Danlos syndrome, limited medical resources

## Abstract

A 1‐year‐old girl from an underserved community presented with irritability, pain, and delayed motor skills. Our genetics outreach program facilitated the diagnosis of Ehlers‐Danlos syndrome masquerading as developmental delay after noting hyperextensible skin. Diagnosis for this family allows for state‐of‐the‐art cardiac monitoring and appropriate symptomatic treatment for this rare disease.

## 1 INTRODUCTION

Exome sequencing has identified *COL5A1*‐related Ehlers‐Danlos syndrome (MIM 130000) in a 15‐month‐old infant who resides in a Caribbean region where genetic services were previously not available. Due to the limited medical resources on the small Caribbean island where this child and her family reside, it is necessary to travel to a larger nearby island to access pediatric care. The parents were concerned about the child's extreme irritability and delayed developmental milestones. Physical examination revealed low muscle tone and diminished reflexes accompanied with failure to sustain eye contact, suggesting a neurological disorder. By fortuitous chance, a visiting clinical geneticist was able to examine the patient and noted velvety, doughy, slightly hyperextensible skin, and joint laxity. Exome sequencing of the family triad revealed that the child carried a novel de novo mutation in the *Collagen, Type V, Alpha 1* (*COL5A1*) gene suggesting Ehlers‐Danlos syndrome (EDS). This diagnosis caused management to focus away from a neurological disorder and instead address issues related to defective collagen production. Now appropriate medical care has been implemented to monitor for possible cardiac manifestations as well as to address the musculoskeletal pain and poor muscle tone associated with this child's EDS.

As permanent and visiting geneticists teaching at a medical school in the Caribbean, we realize that our expertise can be of great benefit to our community. We are assisting local and near by physicians who, because of geographical isolation and disparities in socioeconomic status, are unable to access medical genetic services. Our evolving role gives the physicians of this community the power to initiate appropriate therapies and suggest lifestyle modifications for some of their patients. One successful example of this practice lay with that of a 1‐year‐old baby girl with developmental delay who was suspected to have a form of autism, after exome sequencing received a correct diagnosis and treatment for Ehlers‐Danlos syndrome.

Ehlers‐Danlos syndrome (EDS) is a group of heritable connective tissue disorders caused by a defect in collagen fibrils or collagen‐modifying enzymes.^1^ EDS is genetically and phenotypically heterogeneous with many clinical subtypes. The presentation of classical EDS (types I and the less severe type II, MIM#s 130000 and 130010, respectively) is due to formation of abnormal collagen fibers and includes joint laxity, aortic root dilation, and hyper extensible fragile skin. EDS patients also exhibit poor wound healing and subsequent formation of atrophic scars. In infants, classic EDS may manifest as low muscle tone and delay of developmental milestones such as sitting, standing, and walking.^2^

In the late 1970s, electron microscopy studies of skin biopsies from persons with classical EDS showed a disturbance in heterotypic collagen fibrils made of collagen types I and V. Notably, these fibrils were 13%‐40% larger in diameter with an irregular shape compared to controls.^3^ Almost two decades later, transgenic and linkage analysis studies provided evidence that EDS types I and II are linked to the components of type V collagen, namely *COL5A1* and *COL5A2*.^4^, ^5^, ^6^, ^7^, ^8^, ^9^, ^10^ Quantitatively, type V collagen is a minor fibrillar collagen that is found in a variety of tissues. There are three gene products which can form three different versions of the fibril. The three genes, *COL5A1*,* COL5A2,* and *COL5A3* encode proteins which can form either two heterotrimers ([α1(V)]_2_α2(V) or α1(V)α2(V)α3(V)) or a homotrimer ([α3(V)]_3_). More than half of the patients with classic EDS carry a mutation on either *COL5A1* or *COL5A2* genes. The most common type of mutation introduces a premature stop codon in the mRNA transcript which then leads to halpoinsufficiency of the peptide.^11^

There is no known cure for EDS and therapy is often multi‐layered, based on presenting features. Typically, children with classical EDS receive regular cardiac monitoring by electrocardiogram to monitor for aortic dilation and/or mitral valve prolapse, physiotherapy to assist with hypotonia and motor development, non‐weight‐bearing exercise to promote coordination and muscle strength, advice for treatment of wounds, and help to manage pain.

## 2 MATERIALS AND METHODS

Consent was obtained from the parents of the patient. We used a local consenting form that was constructed under the auspices of our local institutional review board (IRB) to best relate to the local customs of our patients and families. This is a Caribbean community IRB that is registered with the Office of Human Research Protections (OHRP) in the US Department of Health and Human Services, (IRB Registration Number: IRB00010095). All authors received USA National Institutes of Health approved training for human subject research. Additionally, consent was obtained to allow genetic analysis in a Canadian research facility (consent form created in Alberta Children's Hospital).

Whole blood was sent in an insulated container by FedEx to the McGill Group for Suicide Studies at the Douglas Mental Health University Institute. DNA was extracted with the PureGene kit (Qiagen, Hilden, Germany). Sequencing was done using the Agilent SureSelect for ExomeSeq platform on an Illumina HiSeq 2500 to a read depth of 100. Sequence was analyzed using FastQC for quality control. Adapter clipping and read trimming was done with the FASTX‐Toolkit and Trimmomatic. Alignment was accomplished with Bowtie2 software. We used Genome Analysis Toolkit for variant calling.

## 3 CLINICAL REPORT

An Afro‐Caribbean couple sought pediatric medical advice because their 10‐month‐old female infant displayed extreme irritability since birth. The mother remarked that the infant cried excessively especially when being lifted up and her limbs were hypermobile. Despite great effort, mother was often unable to provide comfort to her child. The parents were also concerned that the child was not reaching key developmental milestones. Upon physical examination, the infant was alert, had relatively good neck control but was unable to crawl or sit without support. Head circumference was at the 98th percentile and her ears were low set. It was noted that the girl was not sustaining eye contact and was not cooperative. In addition, vocalization was not developing appropriately for her age. A non‐contrast CT scan was performed which revealed a normal variant of cavum septum pellucidum et vergae which has occasionally been associated with fetal neural maldevelopment. Based on the above features a possible neurological cause for her condition was being discussed.

By fortuitous chance, we organized a meeting between a visiting clinical geneticist, the local pediatrician, and the family when the child was 15 months old. Physical examination of the child showed global low muscle tone and diminished reflexes, low set ears, deformational plagiocephaly, and joint hypermobility. Her right leg had a tendency to cross over the left leg. Bilateral hyperextension of the lower limbs was noted with a more severe hyperextension of the left leg. While trying to grasp an object, the child first flexed her fingers and then flexed the thumb. The thumb did not exhibit a coordinated movement. A slight reduction in Babinski reflexes was noted.

**Figure 1:**
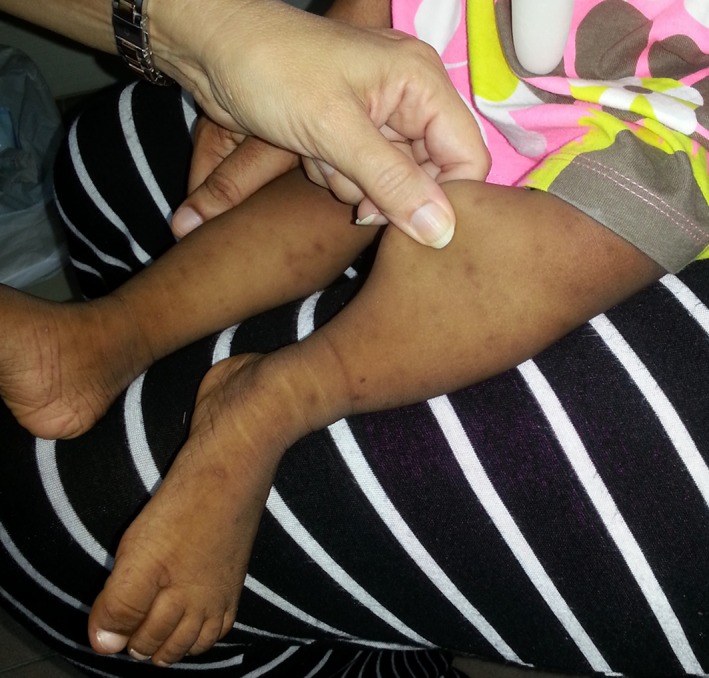
Patient's skin at 15 months old showing characteristic hyperextensibility with a velvety or doughy texture

The child's skin was soft, velvety, and slightly hyper extensible Figure 1. The mother reported that her child had a peculiar breath odor which was reduced during a regimen of antibiotics that was prescribed for an ear infection. Upon completion of the antibiotic treatment, the odor returned. After obtaining consent, a sample of blood was drawn for genetic analysis.

## 4 GENETIC CHARACTERIZATION

The exon region of genes comprises approximately 1% of the entire genome yet the majority of disease‐causing genetic variants are found within this region. Recent innovations to next‐generation sequencing technology have decreased the cost of sequencing while increasing both the speed and accuracy of acquired sequence data. Whole exome sequencing specifically targets the generation of sequence information from only the exon region. Thus, we chose the cost‐effective whole exome sequencing as our first‐tier test.

**Figure 2:**
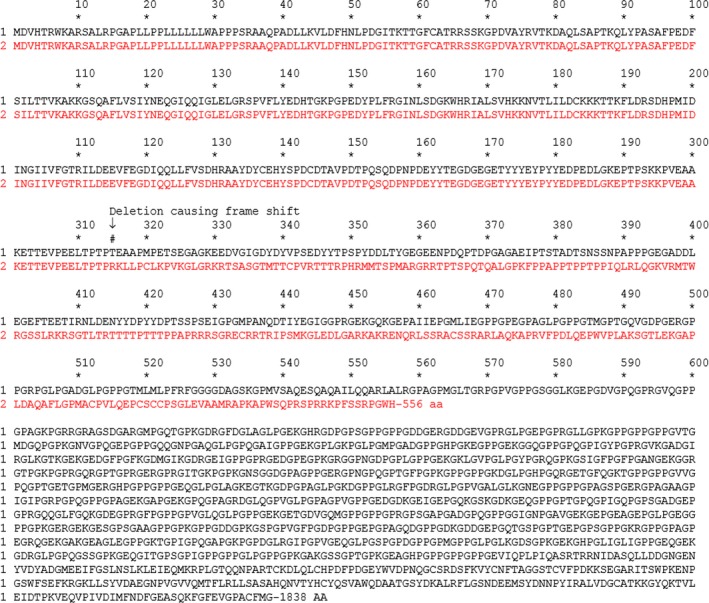
Predicted sequence of the patient's complete primary structure of collagen pro‐α1(V) protein (in red) highlighting the p.Thr315Argfs*242 mutation. Reference sequence, UniProtKB/Swiss‐Prot: P20908.3, is shown in black

Whole exome sequencing revealed a heterozygous deletion of a single cytosine base, NM_000093.4:c.944delC, which is in the seventh exon of the *COL5A1* gene. This deletion is predicted to create a frameshift mutation at amino acid number 315 of the pro‐α1(V) chain. The frameshift would then introduce a premature stop codon resulting in an altered 556 amino acids long pro‐peptide—p.Thr315Argfs*242 Figure 2. The truncated protein would not contain important pro‐peptide C‐terminal cysteine residues required for processing and maturation of type V collagen, likely resulting in halpoinsufficiency of the pro‐α1(V) chain. The mutation was not found in either parent, suggesting a de novo occurrence. A search of ClinVar, ExAC, gnomAD, and LOVD databases indicates that this mutation has yet to be reported.

With this information, our team immediately contacted the family to discuss caring for a child with EDS. We then organized for a charitable organization to perform a baseline echocardiogram and schedule annual cardiac monitoring. The local pediatrician implemented a protocol for the treatment of pain. Our team visited the family 1 year after diagnosis. The characteristic skin hyperextensibility of EDS became more prominent. The parents were happy to report that their daughter appeared to be less irritable, and her development was progressing well. Both the baseline echocardiogram and a follow up 1 year later were unremarkable. At this consultation, the parents expressed appreciation for knowing that their daughter had a condition affecting her connective tissue, and that they need not worry as much about her neurological development.

## 5 DISCUSSION

EDS is a rare disorder and to our knowledge, only one case has been published about this condition in the Caribbean region.^12^ The child described in this case study lives on a sparsely populated island and requires a ferry to access a pediatrician on a neighboring island. One of the pediatric appointments for this child was timed to coincide with a visiting clinical geneticist (WH). During this visit, velvety, doughy skin, and mild joint laxity was observed along with the presenting features of irritability, and slight dysmorphology.

Initially, the family was most concerned about the child's history of an irritable disposition and delayed developmental milestones. At 10 months of age, physical examination showed that the child was alert but did not sustain eye contact. Neurological examination showed low muscle tone and diminished reflexes with a weak Babinski reflex. The medical community and the family began to suspect that the child could have a developmental, behavioral, or neurological disorder. Following the diagnosis of EDS, the family was informed that the constant crying was likely due the baby's discomfort. Numerous reports document chronic pain in patients with EDS.^13^, ^14^, ^15^ Pain might manifest from the reduced collagen deposition in the skin, ligaments, tendons, joints, and bones. There is a growing body of literature which suggests a possible association between connective tissue disorders, such as EDS, and symptoms of autism or psychiatric disorders.^16^, ^17^, ^18^, ^19^ It has further been suggested that intense pain may contribute to the emotional burden of the disorder and that psychiatric features may be secondary to EDS.^20^, ^21^, ^22^ As such, we will continue to monitor the child for autistic and psychiatric symptoms.

The parents of our patient were relieved to have an explanation for their daughter's behavior and that her symptoms can now be addressed. The diagnosis has helped this family plan and prepare for a healthier life that includes managing the disorder in their child.

Importantly, genetic diagnosis allowed us to immediately schedule the baby for a baseline echocardiogram, and ensure yearly monitoring. The parents were advised to monitor their child's physical activities to avoid skin tears. Educational pamphlets were provided to the parents for best practices for suturing of cuts, and treatment of minor wounds. This educational material was then placed into the child's medical file. We were also able to include advice for combined management of pain including analgesics, heat, and physical therapy.^23^, ^24^, ^25^, ^26^, ^27^, ^28^ Lastly, dental problems have been reported for patients with classical EDS,^29^ and the family have been advised as to the importance of good dental hygiene. Special considerations must be made concerning the use of local anesthetics during dental procedures as there is evidence that persons with EDS are often resistant to local anesthetics.^30^^,^ 31

This case illustrates the power of a definitive diagnosis and the importance of genetic services in a resource‐limited community. Here, we describe a pro bono team of clinical geneticists, research geneticists, and the people of a molecular genetics laboratory who had the privilege of assisting a family who hitherto had restricted access to genetic testing.

## CONFLICT OF INTEREST

There are no conflict of interests to disclose by any of the authors.

## AUTHOR CONTRIBUTION

AW & TJ**:** wrote the original manuscript; BN and WAH: assessed the patient and wrote the clinical synopsis; CE and JFT: generated and analyzed the in‐depth exome sequencing; AS: coordinated clinical visits, institutional IRB approval and reviewed the manuscript; MM: edited the manuscript, generated the figures, and supervised the project.
